# Evidence of ecological niche shift in *Rhododendron ponticum* (L.) in Britain: Hybridization as a possible cause of rapid niche expansion

**DOI:** 10.1002/ece3.6036

**Published:** 2020-02-11

**Authors:** Syed Amir Manzoor, Geoffrey Griffiths, Maxwell C. Obiakara, Citlalli Edith Esparza‐Estrada, Martin Lukac

**Affiliations:** ^1^ School of Agriculture, Policy and Development University of Reading Reading UK; ^2^ Department of Forestry & Range Management FAS&T Bahauddin Zakariya University Multan Multan Pakistan; ^3^ Department of Geography and Environmental Science University of Reading Reading UK; ^4^ Department of Botany, Ecology Unit University of Ibadan Ibadan Nigeria; ^5^ Laboratorio de Macroecología Evolutiva, Red de Biología Evolutiva, Instituto de Ecología, A.C. Veracruz México; ^6^ Faculty of Forestry and Wood Sciences Czech University of Life Sciences Prague Prague Czech Republic

**Keywords:** biological invasion, hybridization, invasive species, niche shift, *Rhododendron ponticum*

## Abstract

Biological invasions threaten global biodiversity and natural resources. Anticipating future invasions is central to strategies for combating the spread of invasive species. Ecological niche models are thus increasingly used to predict potential distribution of invasive species. In this study, we compare ecological niches of *Rhododendron ponticum* in its native (Iberian Peninsula) and invasive (Britain) ranges. Here, we test the conservation of ecological niche between invasive and native populations of *R. ponticum* using principal component analysis, niche dynamics analysis, and MaxEnt‐based reciprocal niche modeling. We show that niche overlap between native and invasive populations is very low, leading us to the conclusion that the two niches are not equivalent and are dissimilar. We conclude that *R. ponticum* occupies novel environmental conditions in Britain. However, the evidence of niche shift presented in this study should be treated with caution because of nonanalogue climatic conditions between native and invasive ranges and a small population size in the native range. We then frame our results in the context of contradicting genetic evidence on possible hybridization of this invasive species in Britain. We argue that the existing contradictory studies on whether hybridization caused niche shift in *R. ponticum* are not sufficient to prove or disprove this hypothesis. However, we present a series of theoretical arguments which indicate that hybridization is a likely cause of the observed niche expansion of *R. ponticum* in Britain.

## Graphical abstract

This study presents evidence of niche shift in *Rhododendron ponticum* (L.), an invasive plant species in Britain.

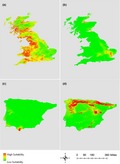

## 1 INTRODUCTION

Uncontrolled biological invasions by plants are among the most severe phenomena attributable to climate change and environmental disturbance by humans (Tingley, Vallinoto, Sequeira, & Kearney, 2014). Invasive species replace native vegetation, which often leads to an alteration of ecosystem structure and function due to the simplification of plant community. Biological invasion may cause a disruption of nutrient cycles, reduction of soil health, and a decrease of net primary productivity (Manzoor, Griffiths, Iizuka, & Lukac, 2018). Understanding the factors that define the geographic distribution of a species, and prevent it from invading other environments, is one of the fundamental goals in ecology. Niche theory suggests that the area occupied by a species is defined by a set of biotic and abiotic factors and dispersal barriers. Successful biological invasions must thus be facilitated by a sequence of events. Breaching a dispersal barrier must be supported by climatic similarity between the native and invasive (also referred to as invaded, introduced, or exotic) ranges and followed by attaining a competitive advantage against native species (Banerjee, Mukherjee, & Dewanji, 2017; Tingley et al., 2014). In most invasive species, however, the relative contribution of these factors to the shaping of range limits is not clearly understood (Tingley et al., 2014). A standard approach to the evaluation of the new niche of an invasive species is to analyze the similarity of environmental conditions between the native and invasive ranges. The correlation between the environment and the observed distribution of species is considered pivotal to species’ introduction, establishment, and expansion in new environments (Jiménez‐Valverde et al., 2011; Pearman, Guisan, Broennimann, & Randin, 2008).

Anticipating the spread of invasive species is central to the creation and application of effective management strategies (Minardo, Heger, Miles, Zipes, & Prystowsky, 1988). Ecological niche models (ENMs) are by far the most widely used predictive tool used to assess the invasiveness of species (Bosso, De Conno, & Russo, 2017; Cunze, Kochmann, Koch, & Klimpel, 2018; Thapa, Chitale, Rijal, Bisht, & Shrestha, 2018). The predictive power of ENMs rests upon the assumption that the relationship between a species and its ecological niche is conservative over space and time (i.e., the fundamental niche remains unchanged or changes very slowly due to evolution) (Huntley, Bartlein, & Prentice, 1989; Pearman et al., 2008; Tingley et al., 2014). This assumption, known as the niche conservatism hypothesis, implies that a species in the invasive range is likely to occupy environmental conditions similar to those typical for its native range (Petitpierre et al., 2012). Modern ENMs, and the increasing availability of species presence data, offer an opportunity to test this hypothesis. This endeavor is interesting for two reasons: (a) A significant violation of niche conservatism in a species is a strong indicator of adaptive (Klironomos, 2002) or evolutionary (Lavergne & Molofsky, 2007) changes during the invasion and (b) in an era of rapid environmental change, improved understanding of if and when an environment suitable for an invasion will appear may be crucial to ecosystem management and conservation.

Recent studies on niche conservatism of species report conflicting results. While there is a plethora of evidence confirming spatial and temporal niche conservatism (Petitpierre et al., 2012), some insects (Fitzpatrick, Weltzin, Sanders, & Dunn, 2007), plants (Gallagher, Beaumont, Hughes, & Leishman, 2010), and fish (Lauzeral et al., 2011) were shown not to conform with the theory. A mismatch between the native and the invasive environmental niche can signify a shift in the fundamental niche (“the requirements of a species to maintain a positive population growth rate, disregarding biotic interactions” (Guisan, Petitpierre, Broennimann, Daehler, & Kueffer, 2014)), the potential niche (“the subset of fundamental niche nonrestricted by biotic interactions that corresponds to combinations of environmental variables at a given time, susceptible to variations due to environmental changes that may lead niche shifts in shape, size or position in environmental space” (Jackson & Overpeck, 2000)), or the realized niche (“the portion of the fundamental niche in which a species has positive population growth rates, given the constraining effects of biological interactions such as competition” (Guisan et al., 2014; Pearman et al., 2008)). Thus, a shift in the fundamental niche may be a consequence of evolutionary change (i.e., genetic drift and/or hybridization; Gallagher et al., 2010). A shift in the realized niche as a subset of potential niche may then be attributable to the availability of unoccupied niches in the invasive range or to a release from top–down regulators due to their absence in the new environment (i.e., predators or pathogens; Broennimann et al., 2007).

Assuming that an invasive species occupies all suitable conditions in its native range, any difference between its niche in the native and invasive ranges can be due to three distinct processes: niche expansion (invasive species occupies new environmental conditions in the invasive range compared with its native range), niche unfilling (partial filling of the native niche in the invasive range; Petitpierre et al., 2012), and niche stability (proportion of native niche of an invasive species overlapping with its invasive niche; Pearman et al., 2008). Two different hypotheses need to be tested to determine which of these processes drives niche differentiation for a given invasive species, niche equivalency (native and non‐native niches are indistinguishable and interchangeable) and niche similarity (native and invasive niches are more similar than expected by chance; Strubbe, Beauchard, & Matthysen, 2015; Warren, Glor, & Turelli, 2008).

Biological invasion may also lead to hybridization if similar, but historically isolated, taxa come into contact. Hybridization of invasive species can produce genetically superior populations (Blaine Marchant, Soltis, & Soltis, 2016; Dietz & Edwards, 2006; Molina‐Henao & Hopkins, 2019) which are potentially better at exploiting new environmental conditions as compared to their parents (Sheth & Angert, 2014). Undetected hybridization of closely related species may thus lead to an apparent shift in fundamental niche of either or both parent species (Parisod & Broennimann, 2016). Despite the potentially large impact of hybridization on the rapid evolution of new species, to date only a limited number of investigations have explored the role of hybridization in niche occupancy (Molina‐Henao & Hopkins, 2019; Mukherjee et al., 2012).

In Great Britain, *Rhododendron ponticum* (L.) is a classic example of an invasive species that has spread at a massive scale and caused significant environmental and economic damage (Jackson, 2008). *Rhododendron ponticum* was introduced to the British Isles as an ornamental plant from mainland Europe in the eighteenth century. It is a perennial, evergreen shrub that generally invades woodlands (Tiedeken & Stout, 2015), although it is known to colonize other types of habitats too (Manzoor, Griffiths, Iizuka, et al., 2018). The success of the invasion of *R. ponticum* in Britain is attributed to its ecological and biological characteristics; it produces copious amounts of seeds and can tolerate shaded and nutrient‐depleted soils (Dehnen‐Schmutz & Williamson, 2006). Its growth prevents germination of native plant species by releasing allelochemicals into the soil (Cross, 1981; Stephenson, MacKenzie, Edwards, & Travis, 2006).

The suitability of *R. ponticum* to the British environment and its invasiveness were first thought to result from a hybridization of *R. ponticum* with *Rhododendron catawbiense*, (a North American species), a process which supposedly lent frost hardiness to the British *R. ponticum* population (Milne & Abbott, 2000). However, this thesis was later rejected by other reports which did not find any genetic evidence of such hybridization (Erfmeier, Tsaliki, Roß, & Bruelheide, 2011). The spread of *R. ponticum* thus represents an opportunity to test how the current niche occupied in Britain corresponds to that in its native Iberia. To evaluate this, we tested two different hypotheses: (a) The native and invaded niches are equivalent (native and invasive niches are interchangeable), and (b) the native and invaded niches are similar (the native and invasive niches are more similar than expected by chance). Thus, in this study we examine the environmental niche of *R. ponticum* in its invasive and native ranges and interpret our findings in the context of contradicting reports of past hybridization of the British population of *R. ponticum.*

## 2 MATERIALS AND METHODS

### 2.1 Species description and geographic ranges

The native range of *R. ponticum* covers the southern reaches of Spain, western Portugal, and Georgia (Milne & Abbott, 2000). However, the main ancestor of *Rhododendron* in Britain is reported to be the population of *R. ponticum* resident in southern Spain and western Portugal (Dehnen‐Schmutz & Williamson, 2006). We thus consider the Iberian Peninsula as the native range *R ponticum*. We obtained distribution records of this species from the Global Biodiversity Information Facility (http://www.gbif.org/) using the dismo R package. These records were then complemented by a selection of background points (i.e., set of randomly sampled geographical locations that represent the environmental conditions available to an organism (Obiakara & Fourcade, 2018)). These points must cover the area where the species can easily disperse. We defined accessible area for *R. ponticum* as the set of terrestrial ecoregions (Olson et al., 2001) occupied by the species´ distribution, by considering potentially accessible areas and their complete occurrences which allows a reliable estimation of niche overlap.

### 2.2 Variable selection

Ideally, the selection of predictor variables in ENMs should take into account the ecological requirements of the species under investigation (Manzoor, Griffiths, & Lukac, 2018). However, like most invasive species, *R. ponticum* does not lend itself to the provision of autecological information, which makes it difficult to a priori select a specific set of biologically relevant predictors. Following a detailed literature review, we chose 19 bioclimatic variables to model the distribution of *R. ponticum* (Manzoor, Griffiths, Iizuka, et al., 2018). Current (1960–2000) climate data were downloaded from the WorldClim database (Fick & Hijmans, 2017) at a resolution of 30 arc‐seconds (ca. 1 km^2^), which was shown to provide optimal prediction of *R. ponticum* distribution (Manzoor, Griffiths, Iizuka, et al., 2018). The WorldClim (version 2.0) database consists of 19 derived bioclimatic variables that represent climate average, extremes and variability.

Collinearity among predictor variables negatively impacts the model due to the substantial amount of information shared between variables, making it difficult to correctly interpret the relative contribution or importance of variables to model predictions (Dormann et al., 2013). Pearson correlation coefficient cutoff of −.75 ≤ *r* ≤ .75 was applied to select bioclimatic variables to be used in the final model runs (Dormann et al., 2013), chiefly to reduce multicollinearity and to conform to statistical assumptions (Syfert, Smith, & Coomes, 2013). Consequently, we selected four variables: annual mean temperature (Bio 1), minimum temperature of the coldest month (Bio 6), annual mean precipitation (Bio 12), and precipitation of the coldest month (Bio 14) for this study.

### 2.3 Climatic niche analysis

The principal component analysis (PCA‐env) approach proposed by Broennimann (Broennimann et al., 2012) was used to visualize the native and invaded niches of *R. ponticum* in a 2D climatic space. The PCA‐env method compares the environmental conditions available for a species within the study area (background) with observed occurrences and calculates available environmental space defined by the first two axes from the PCA‐env. This method corrects for sampling bias using a smooth kernel density function (Broennimann et al., 2012). We estimated niche overlap between the two geographical ranges using Schoener's *D* index (Schoener, 1970), which ranges from 0 to 1 (i.e., no to complete niche overlap). Niche shift was statistically tested as described by Broennimann et al. (2012).

#### 2.3.1 Test of Niche equivalence and Niche overlap

The niche equivalence test, as initially proposed by Graham, Ron, Santos, Schneider, and Moritz (2004), asks whether niches under comparison are indistinguishable from each other. For this test, the occurrence points from both ranges were pooled together and then randomly split into two sets, maintaining the actual number of occurrences in each range. Niche similarity was calculated following Warren et al. (2008) using two metrics of niche overlap, namely Schoener's *D* (Schoener, 1968) and Warren et al.’s *I* (Warren et al., 2008). These statistics quantify niche overlap and range from 0 (no overlap) to 1 (complete overlap). Niche equivalence was determined by comparing the observed niche overlap values (*D* and *I*) to a null distribution of 1,000 overlap values. Rejection of the niche equivalency hypothesis means that native and invaded niches are not environmentally identical (i.e., not equivalent) (Strubbe et al., 2015). Thus, if the value of the observed niche overlap falls outside the 95% confidence intervals of the null distribution, the null hypothesis of equivalency is rejected (Fick & Hijmans, 2017).

The niche similarity test examines whether the overlap between the observed native and invaded niches of *R. ponticum* is different from the overlap between the observed niche in one range and a randomly selected niche in the other range (based on 1,000 repetitions). Rejection of the niche similarity hypothesis means that overlap of native and non‐native niches is larger than random expectation, that is, the environmental conditions occupied by the species in the non‐native range are more similar to the conditions occupied in the native range than would be expected by chance (Strubbe et al., 2015). Thus, for the niche similarity test, a *p*‐value >.05 is considered to indicate that niches are less similar than expected by chance. Niche similarity test was used in the current study to estimate niche divergence. Niche overlap value above 95% confidence interval of the null hypothesis indicates niche divergence (Fick & Hijmans, 2017).

Niche equivalence and similarity tests only verify whether niche shifts have occurred but do not address their causal mechanism (Sales et al., 2017). To understand *R. ponticum *invasion process, we disentangled niche changes into the processes of niche stability (*S,* the overlap of invaded niche with native niche), unfilling (*U,* the nonoverlapping part of native niche in the invaded niche), and expansion (*E,* the nonoverlapping part of the invaded niche in the native niche) as described by (Pearman et al., 2008). All analyses were carried out in the statistical software R, version 3.1.3 using the ecospat package (Di Cola et al., 2017).

### 2.4 Reciprocal distribution modeling

We used the reciprocal distribution modeling approach (Fitzpatrick et al., 2007) to estimate the potential distribution of *R. ponticum* in its invaded range. Following this approach, a model was first calibrated in the native range (Iberian Peninsula) and projected onto the invaded range (Great Britain). Then, a second model was calibrated in the invaded range and projected onto the native range. Furthermore, models trained in native and invasive ranges predicted habitat suitability in the same ranges as well. The degree of similarity between juxtaposed (calibrated in the other range) and local models (calibrated in the same range) was assessed.

We used MaxEnt (3.4.1), a maximum entropy‐based machine learning (presence/background) algorithm for distribution modeling. MaxEnt predicts the probability distribution of a species on the basis of a given set of predictor variables and presence‐only species occurrence data (Phillips, Dudik, & Schapire, 2004). We spatially rarefied the presence points acquired from GBIF by excluding all but one point within 1 km^2^ which gave us a reasonably large sample size (79 in the Iberian Peninsula and 6579 in Britain). We applied the recommended screening and verification of occurrence records (Manzoor, Griffiths, & Lukac, 2018; Wisz et al., 2008). The complementary log‐log output of MaxEnt was used to produce an estimate of occurrence probability for each model.

#### 2.4.1 Model complexity and tuning

Various studies have confirmed that calibrating MaxEnt models with default settings frequently leads to highly complex models, a case‐specific tuning of the model is thus recommended (Moreno‐Amat et al., 2015) (for details see (Manzoor, Griffiths, & Lukac, 2018)) To select the modeling parameters which give the best trade‐off between model performance and complexity, we used ENMeval (Muscarella et al., 2014) to build models with all possible combinations of these parameters. We produced a total of 48 models using six combination of these feature classes (L, H, LQ, LQH, LQHP, LQHPT) and eight regularization multipliers (0.5, 1.0, 1.5, 2.0, 2.5, 3.0, 3.5, 4.0) (Obiakara & Fourcade, 2018). All models were built using 10,000 background points randomly selected within the calibration area (Iberian Peninsula for the model calibrated with native records and Great Britain for the model calibrated with invasive records). We used the “block” method implemented in ENMeval to partition data into four geographically distinct calibration and evaluation datasets, in order to conduct spatially independent tests of model performance. Finally, we selected the most suitable model using the Akaike information criterion corrected for small sample sizes (ΔAICc < 2) (Boyce, Vernier, Nielsen, & Schmiegelow, 2002). The final model used included LQHP feature classes and a regularization multiplier of 0.5.

#### 2.4.2 Model evaluation

Area under the ROC (receiver operating characteristic) curve (AUC) was used to test the performance of the model against actual observations in both the ranges (Elith et al., 2006). An AUC value of 0.5 shows that the model does not predict any better than random chance, whereas a value closer to 1 indicates a better performance of the model (Swets, 1988). Percentage of contribution and permutation importance contribution were used to assess the relative significance of predictor variables. In addition to AUC, we used continuous Boyce index (CBI) as an additional assessment tool. The Boyce index requires presence data only and measures by how much model predictions differ from a random distribution of observed presence across the prediction gradient (for details, see Manzoor, Griffiths, & Lukac, 2018). The continuous values of the Boyce index vary between −1 and +1. Positive values indicate a model where predictions are consistent with the distribution of actual presence data, values close to zero mean that the model is no different from a random model and negative values indicate counter predictions (e.g., predicting no occurrence in areas where actual presence is recorded) (Boyce et al., 2002; Hirzel, Le Lay, Helfer, Randin, & Guisan, 2006). We also used the true skill statistic (TSS) as an additional accuracy evaluation measure (Allouche, Tsoar, & Kadmon, 2006). The value of TSS ranges from −1 to +1. Values above zero indicate better model performance than chance alone.

## 3 RESULTS

**Figure 1:**
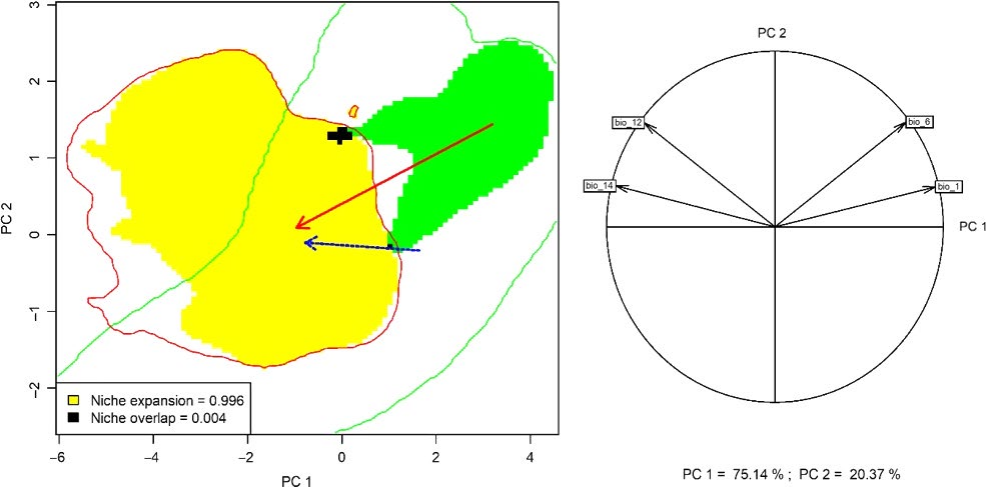
(a) Principal component analysis of niche shift of *Rhododendron ponticum*. Green and red contour lines demarcate available niche in the native (Iberia) and invaded (GB) ranges, the blue arrow indicates a shift of the centroid of available niche. Green and yellow areas represent occupied niches in the native and invasive ranges, respectively. The red arrow links the centroid of the native and invasive distribution. (b) Correlation circle indicates the weight of each variable on the niche space as defined by the first two principal component axes

**Figure 2:**
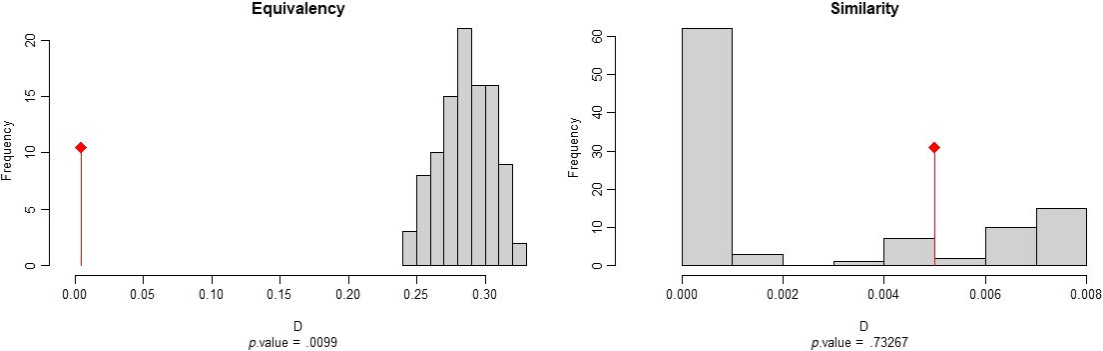
Niche equivalency and similarity tests comparing native and invaded ranges of *Rhododendron ponticum*. Schoener's *D* index on *x*‐axis indicates none (*D* = 0) to complete (*D* = 1) niche overlap. Red lines show overlap observed in this study, gray bars show a simulated null distribution of 1,000 random replicates

**Figure 3:**
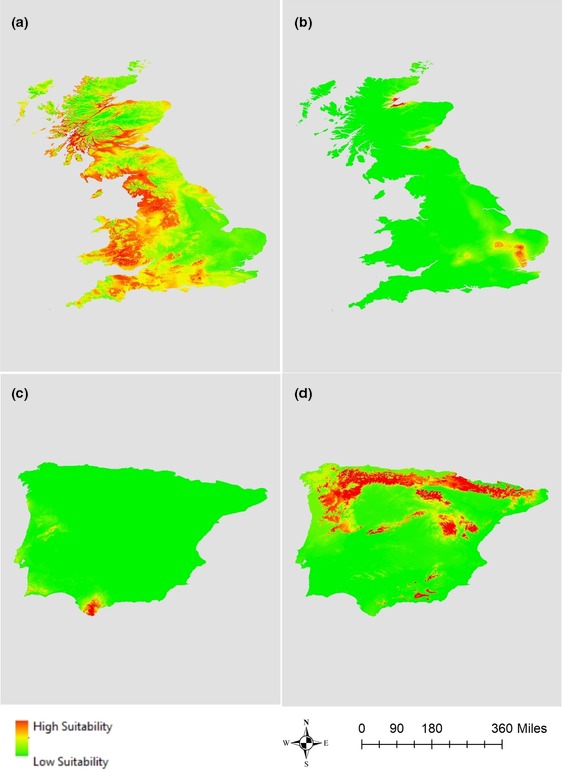
Distribution maps of *Rhododendron ponticum* in Britain based on models calibrated in Britain (a) and the reciprocal model trained in the Iberian Peninsula (b). Distribution maps of *R. ponticum* in the Iberian Peninsula based on a model trained in the Iberian Peninsula (c) and the reciprocal model trained in Britain (d)

The climatic space occupied by *R. ponticum* in its native and invaded ranges is represented in Figure 1. The correlation circle in pane b shows that the first two PCA axes explained 96.29% of the variance in the set of four predictor variables. Annual mean temperature (Bio 1) and annual mean precipitation (Bio 12) were the most important variables in the first and second principal components. Niche overlap of *R. ponticum* between the Iberian Peninsula and Great Britain was very low (Schoener's *D* = 0.005, Warren's *I* = 0.004), following the classification scheme of Rödder and Engler (2011). The test of equivalence between native and invaded realized niches of *R. ponticum* showed statistically significant differences, which are clearly visible in Figure 2. We therefore reject the null hypothesis of niche equivalency and accept the alternative that the niches are ecologically distinct (Guisan et al., 2014). In addition, the similarity test results in a nonsignificant value of *D* and *I*, suggesting that the two niches are less similar than random chance (Table 1). In other words, environmental conditions occupied by the species in the invasive range are less similar to conditions occupied in the native range than would be expected by chance. Furthermore, given the low niche overlap and high expansion values (*E = *0.996), niche dynamics test suggests that *R. ponticum* currently occupies new environment in Britain. Similarly, a very high unfilling (*U*) value indicates that the conditions occupied by the species in the native range are still unoccupied in the invasive range. Furthermore, the results of the reciprocal distribution modeling suggest that the model calibrated in the native range failed to predict occurrence in the invasive range with reasonable accuracy, and vice versa (Figure 3). The models calibrated in the same range, however, predicted the distribution of *R. ponticum* reasonably well. The AUC, Boyce index, and TSS values for all combinations of projections are presented in Table 2.

**Table 1:** Schoener's *D* values indicate niche overlap (corresponding *p* values show statistical significance)

Equivalence	Similarity		Expansion	Stability	Unfilling
	Native ‐> Invasive	Invasive ‐> Native			
*D* = 0.005, *p = .009*	*D* = 0.005, *p* = .732	*D* = 0.005, *p* = .151	0.9996	0.0003	0.9964
*I* = *0.004, p* = .009	*I* = 0.004, *p* = .742	*I* = 0.004, *p* = .93			

Note

Expansion and stability are proportions of nonoverlapping and overlapping invasive niche compared with the native niche, respectively. Unfilling represents the proportion of the native niche available, but not occupied in the exotic niche.

**Table 2:** AUC, Boyce index and true statistical skills (TSS) values indicating the accuracy of models

	Native ‐> Native	Native ‐> Invasive	Invasive ‐> Invasive	Invasive ‐> Native
AUC	0.952	0.4	0.7	0.52
Boyce index	0.94	0.52	0.81	0.3
TSS	0.94	−0.5	0.90	−0.5

## 4 DISCUSSION

In this study, we compared the ecological niche of *R. ponticum* in its native and invasive ranges and tested the hypothesis of niche divergence. While previous studies have analyzed the genetic material of *R. ponticum* populations to look for evidence of hybridization (Erfmeier et al., 2011), our study is the first effort to model ecological niches and spatial distribution of this species in its native and invasive ranges by comparing niche differences in a gridded environmental space. We found a very limited niche overlap between *R. ponticum* populations in the Iberian Peninsula and Britain, as indicated by very high values of niche expansion and niche unfilling. Our results indicate that *R. ponticum* largely occupies novel niches in Britain. Conversely, large values of unfilling (*U*) indicate that a large proportion of environmental conditions occupied in the native niche is available, but unoccupied in the invasive range (Guisan et al., 2014). The results of niche equivalency and niche similarity tests show that the two niches are not equivalent and that the ecological niche of *R. ponticum* has shifted in the invasive range (i.e., the native niche is not conserved). MaxEnt‐based reciprocal distribution models fail to predict species distribution in juxtaposed target ranges, confirming the earlier finding that environmental conditions occupied by *R. ponticum* in its native range differ from those occupied in the invasive range.

The pattern and the extent to which species’ niches are conserved or shift over space and time is a key determinant of their response to local and global environmental change (Thornton & Murray, 2014). Predictive maps generated by reciprocal distribution models suggest that the current distribution of *R. ponticum* in Britain is mostly clustered in the western and northern parts which are the cooler and more humid parts of the country. In its native range, the species is present at the southern tip of Spain and southern and western parts of Portugal, typical for milder temperature regime and less rainfall. Thus, the distribution model calibrated in Iberia incorrectly places the distribution to eastern Britain. This part of Britain is ecologically similar to conditions occupied by the species in the Iberian Peninsula, but it is not where the species is currently found. The actual distribution of *R. ponticum* in Britain is centered on Wales and the Scottish uplands, areas with some of the lowest temperatures in the whole of Britain. This mismatch also explains the high value of niche expansion (*E* = 0.996), suggesting that *R. ponticum* in Britain is present in locations where the environmental conditions are very different from those of its native range on the Iberian Peninsula. This type of invasive behavior is very similar to that reported by a remarkable number of studies where hybridization fostered the emergence of successful invasive populations (Arrigo et al., 2016; Ellstrand & Schierenbeck, 2000).

Although niche shift in terrestrial plants is rare (Petitpierre et al., 2012), even large shifts in environmental niches such as the one observed in our study could potentially be a result of evolutionary changes. Hybridization of the invading species with a local or another introduced species has been shown to produce superior adaptive traits (Ellstrand & Schierenbeck, 2000). For example, the abundance of *R. ponticum* in Wales and Scotland is often attributed to the frost hardiness adaptation of this species, which, in turn, is considered a consequence of hybridization. Therefore, how significant a role does hybridization play in successful invasion and divergence of ecological niches?

### 4.1 The role of hybridization in niche expansion

Ecological niche expansion is often, but not exclusively, associated with hybridization (Hedrick, 2013). This thesis rests on the proposition that under continuous presence of barriers to natural dispersal and establishment (e.g., climate, topography, predators, or competitors (Nasiri, Darvishsefat, Rafiee, Shirvany, & Hemat, 2018)), it is the species that must change if it is to expand its range. Populations of a species that inhabit the leading edge of an expansion are likely to need genetic adaptations driving the colonization of new environments, currently denied to them by barriers to dispersal (Barrett & Schluter, 2008; Sexton, McIntyre, Angert, & Rice, 2009). One way to gain such adaptations is genetic mutations, followed by subsequent selection; however, in most species, this process operates at long timescales—too long to explain the speed of observed biological invasions (Orr & Unckless, 2008; Phillips, 1996). Alternatively, the population of a species at the leading edge may hybridize with an established native species or another introduced species to produce an advantageous combination of traits (Hedrick, 2013) and thus sustain the expanding population until new adaptive traits arise due to mutation (Drake, 2006). Theoretically, a species may overcome its dispersal barriers to expand its geographic range by occupying microenvironments to which its ancestral populations are already adapted to certain extent (Early & Sax, 2014). However, this type of expansion is be different from that driven by hybridization. A key implications of the “hybridization facilitates niche expansion” hypothesis is that the hybridizing population expands to environmental conditions where the native population does not occur (Pfennig, Kelly, & Pierce, 2016). Thus, rapid expansion into novel environments is a strong indication of hybridization.

### 4.2 Niche expansion of *R. ponticum* in Great Britain—evidence of possible hybridization

An early study of hybridization of *R. ponticum* in Britain reported on 260 naturalized accessions of *R. ponticum* and presented evidence that 89% of those accessions possessed a cpDNA haplotype occurring in the Spanish population of *R. ponticum*, while 10% of accessions had a haplotype unique to the Portuguese material (Milne & Abbott, 2000). Furthermore, rDNA or cpDNA evidence of hybridization from *R. catawbiense*—which is native to North America—was found only in 27 British accessions of *R. ponticum*. Interestingly, these 27 accessions were significantly more abundant in Britain's coldest regions. Since *R. catawbiense* is more resistant to frost than *R. potnicum*, the conclusion that suggests itself is that *R. ponticum* had acquired frost resistance genes from *R. catawbiencei*, leading to the expansion of its range into the western and north‐western parts of Britain (Erfmeier & Bruelheide, 2004).

Our study, although focusing on climatic factors only and ignoring other critical ecological components such as interspecific competition, soil, and land cover composition, is compatible with the hypothesis that hybridization contributed to invasiveness of *R. ponticum* in the colder regions of Britain (Arrigo et al., 2016; Ellstrand & Schierenbeck, 2000).

### 4.3 Hybridization *may not* be the cause of niche expansion—a counter‐narrative

A decade after the original “*R. ponticum* x *R. catawbiense”* hybridization explanation for the successful colonization of western Britain by *R. ponticum,* it was challenged by a study which sampled populations in Ireland. The researchers used more advanced genetic analysis techniques and concluded that there is no evidence of Irish *R. ponticum* sharing either ecological or morphological traits with North American *R. catawbiense* (Erfmeier et al., 2011). Amplified Fragment Length Polymorphism (AFLP) data confirmed the distinctiveness of *R. ponticum* from its North American relative, leading the authors to reject the hybridization hypothesis presented in the earlier report (Milne & Abbott, 2000). Interestingly, increased frost tolerance—which the former study presented as evidence of hybridization—was also found in the Irish *R. ponticum* populations where the temperatures are mild, and therefore, such a trait does not seem to have an adaptive value (Erfmeier et al., 2011).

There is a plethora of evidence that hybridization and expansion into novel environments are strongly correlated; however, hybridization may not be the driver of niche expansion in some cases (Currat, Ruedi, Petit, & Excoffier, 2008; Melo‐Ferreira et al., 2007; Orozco‐Terwengel, Andreone, Louis, & Vences, 2013). The mere observation that a species has expanded its range into novel environments is not sufficient to claim that the range expansion was enabled by hybridization. Unless backed up with consolidated empirical evidence from genetic investigations of the native and invasive populations, it may not be correct to claim that niche expansion is explained by hybridization (Pfennig et al., 2016).

### 4.4 The curious case of *R. ponticum* niche expansion

We have shown that the native and invaded niches of *R. ponticum* are not equivalent and that they are less similar than random chance (Figure 2). Our analysis clearly shows that the population of *R. ponticum* in Britain has expanded and shifted its range to such an extent that, using a model trained on its native range to predict it, results in a complete mismatch. Given that the species was brought to the country about 200 years ago, such a shift would indicate genetic change caused by a rapid introgression of genes rather than mutation. Both existing reports on the genetics of *R. ponticum* in Britain have limitations. Milne and Abbott (2000) posit that increased frost hardiness results from directional selection introgression, but were limited by the lack of sufficient genetic analysis. The follow‐up study of Erfmeier et al. (2011) was limited only to the Irish population of *R. ponticum* and thus may not be generalizable to the Welsh and Scottish populations. Only a concerted testing of both an introgression by means of nuclear markers and the frost hardiness by means of experimental determination on a sample covering all populations from the British Isles may be able to identify the driver of *R. ponticum* expansion in Britain (Erfmeier et al., 2011). It is essential to keep in sight that the observed niche shift could either be due to an evolutionary process such as hybridization (changing fundamental niche hypothesis (Blossey & Notzold, 1995)) or it could be driven by a difference in the biotic environment between the native and invasive range (enemy‐release hypothesis (Dietz & Edwards, 2006)), or indeed due to a combination of these reasons.

Our niche shift and reciprocal modeling indicate a definitive shift in the environmental adaptation of *R. ponticum* in its invasive range, but a more comprehensive modeling approach using a wider set of environmental variables may be able to test causality rather than correlation. A future niche shift modeling exercise may combine data describing *R. ponticum* populations from North America and Iberian Peninsula (as native ranges) to predict observed niche shift. We based our analysis on the climatic factors only; however, it is conceivable that in some instances, these may only partially explain observed niche shift phenomena. Other, more pertinent nonclimatic factors such as soil properties, land cover, or land use type, may play a more decisive role in explaining niche dynamics (Broennimann et al., 2007). Niche shift analysis is also sensitive to sample size. In our case, the sample describing the presence of *R. ponticum* in the Iberian Peninsula was small relatively to that describing Britain (although still the most comprehensive dataset available for Iberia).

## 5 CONCLUSION

Our study documents a substantial niche shift of *R. ponticum* in Great Britain. We show that in Britain, the species occupies a niche that is entirely different from that in its native Iberia, both in terms of equivalence and similarity. On the basis of presented evidence, we believe that hybridization has driven the niche shift of *R. ponticum* in Britain, although we are not able to prove it conclusively. Observed expansion of species range may have been caused by biotic or abiotic factors not considered here. We suggest that a more comprehensive genetic analysis of *R. ponticum* populations in England, Scotland, and Wales is needed to investigate any evidence of hybridization. Future development of ecological niche models that include a mechanistic approach for the species should be considered in order to study more accurately the niche differentiation of the species by hybridization and invasion.

## CONFLICT OF INTEREST

None declared.

## AUTHOR CONTRIBUTION

S.A.M. conducted the niche shift analyses and conceived the manuscript; G.G., M.C.O., and C.E.E.E. assisted with ecological niche modeling and writing; M.L. conceived the manuscript, assisted with writing and editing the manuscript.

## Data Availability

All the data used in this study were retrieved from online open access sources and online links to all sources have been provided within the manuscript.
